# Linking Enzymatic Oxidative Degradation of Lignin to Organics Detoxification

**DOI:** 10.3390/ijms19113373

**Published:** 2018-10-28

**Authors:** Xiaolu Wang, Bin Yao, Xiaoyun Su

**Affiliations:** Key Laboratory for Feed Biotechnology of the Ministry of Agriculture, Feed Research Institute, Chinese Academy of Agricultural Sciences, Beijing 100081, China; xiaolu4444@126.com

**Keywords:** laccase, manganese peroxidase (MnP), lignin peroxidase (LiP), versatile peroxidase (VP), dye-decolorization peroxidase (DyP), lignin, detoxification

## Abstract

The major enzymes involved in lignin degradation are laccase, class II peroxidases (lignin peroxidase, manganese peroxidase, and versatile peroxidase) and dye peroxidase, which use an oxidative or peroxidative mechanism to deconstruct the complex and recalcitrant lignin. Laccase and manganese peroxidase directly oxidize phenolic lignin components, while lignin peroxidase and versatile peroxidase can act on the more recalcitrant non-phenolic lignin compounds. Mediators or co-oxidants not only increase the catalytic ability of these enzymes, but also largely expand their substrate scope to those with higher redox potential or more complicated structures. Neither laccase nor the peroxidases are stringently selective of substrates. The promiscuous nature in substrate preference can be employed in detoxification of a range of organics.

## 1. Introduction

Cellulose, hemicellulose, and lignin are the three major constituents of plants’ cell wall. By intertwining hemicellulose, lignin covers cellulose and they collectively form a recalcitrant barrier, protecting the interior cellular components. Despite the recent discovery of lytic polysaccharide monooxygenases classified in the CAZy (Carbohydrate-Active enzyme) auxiliary activity families (AA) 9, 10, 11, and 13 (http://www.cazy.org), enzymatic degradation of cellulose and hemicellulose is still regarded to be mainly by hydrolysis through the action of glycoside hydrolases. In contrast, deconstruction of lignin depends much on enzymatic oxidation rather than hydrolysis. In addition to those well-characterized or reported, there are many putative ligninolytic enzymes widely distributed in nature belonging to different categories [1]. These enzymes are laccase and peroxidases, which are involved in lignin oxidative degradation, with the latter including lignin peroxidase (LiP), manganese peroxidase (MnP), versatile peroxidase (VP), and dye-decolorization peroxidase (DyP).

The components of lignin are diversified, with structurally divergent *p*-coumaryl alcohol, coniferyl alcohol, sinapyl alcohol, and their methoxylates serving as monomer constituents [2]. Roughly 10% of lignin is phenolic in nature while the rest and major part is non-phenolic. Laccase and MnP directly oxidize phenolic lignin components, while LiP and VP can act on the more recalcitrant non-phenolic lignin compounds.

Neither laccase nor the peroxidases are stringently selective of substrates. The evolved wide substrate specificity enables them to attack the much differing lignin components. With the aid of small molecule mediators, their substrate scope is further broadened. Interestingly, it has long been noticed that some environmental pollutants or xenobiotic compounds, such as estrogenic biphenol A and aflatoxins, have similar structures with those of lignin monomers, raising interest of utilizing lignin oxidative enzymes for detoxification of these compounds. Starting from initial successes, lignin oxidizing enzymes have been rapidly expanded for use in detoxification of organics with much differing structures. Here, we review the impressive achievements in connecting enzymatic oxidative degradation of lignin to detoxification of organics.

## 2. Enzymatic Oxidative Degradation of Lignin

### 2.1. Laccase

Laccase (EC1.10.3.2) is an extracellular copper-containing polyphenol oxidase, with a molecular mass ranging from 58–90 kDa [3,4,5], that catalyze the oxidation of various aromatic compounds, particularly those with electron-donating groups, such as phenols (−OH) [6]. It was first discovered in the Japanese lacquer tree and then also in other plants [7]. Also widely distributed in fungi, such as *Trametes* spp. and *Pleurotus ostreatus*, laccase is the most intensively studied enzyme oxidizing lignin [8]. So far, we have known that the genomes of many ascomycetes and basidiomycetes encode laccase genes.

Laccase contains four copper-binding conserved domains: Cu I (with the motif NH_2_-HXHG-COOH), Cu II (NH_2_-HXH-COOH), Cu III (NH_2_-HXXHXH-COOH), and Cu IV (NH_2_-HCHXXXHXXXXM/L/F-COOH) [9]. The four copper atoms in laccase can be classified into three types, with one being Type I (T1), one being Type II (T2), and two being Type III (T3). Laccase catalyzes four-electron substrate oxidation, resulting in reductive cleavage of a dioxygen bond. The copper atoms within the enzymes play key roles in reduction of O_2_ to H_2_O. For phenolic compounds’ oxidation, the substrate is first oxidized at the T1 copper site. The extracted electron is passed approximately 13 Å through the His-Cys-His pathway to the tri-nuclear copper cluster formed by the T2 and T3 coppers. Ultimately, oxygen is reduced to water by accepting the electron from the trinuclear center [10].

The enzyme exhibits a broad substrate range, oxidizing polyphenols, methoxy substituted phenols, aromatic diamines, and a range of other compounds. Since the redox potential of laccase is low, while phenolic lignin can be directly oxidized by laccase, non-phenolic lignin with high redox potential can only be oxidized by laccases in the presence of an appropriate mediator, such as ABTS (2, 2′-Azino-bis(3-ethylbenzothiazoline-6-sulfonic acid)) or methyl syringate. Normally, the mediator (or co-oxidant) is itself a substrate of laccase. Once it is oxidized by laccase, it can diffuse and act on the high redox potential lignin or those compounds that do not fit the binding pocket of laccase.

### 2.2. Manganese Peroxidase

Manganaese peroxidases (EC 1.11.1.13) are extracellular glycosylated proteins with molecular masses ranging from 32–62.5 kDa, belonging to the family of oxidoreductases [11]. The first MnP was discovered in *Phanerochaete chrysosporium* in the mid-1980s [12]. A heme prosthetic group acts as the co-factor of the MnP enzyme and MnP uses hydrogen peroxide as an oxidant to convert Mn^2+^ into Mn^3+^. In the first step of catalysis, H_2_O_2_ (or a peroxide) enters the active site of MnP. The oxygen atom in H_2_O_2_ binds to the Fe^3+^ in the heme cofactor to form an iron peroxide complex. Two electrons are then transferred from Fe^3+^ to peroxide, leading to breakage of the oxygen-peroxide bond and formation of an Fe^4+^ oxo-porphyrin radical complex. This oxidized intermediate complex is referred to as an MnP compound I. This compound I then binds to a monochelated Mn^2+^, which, by donating an electron, quench the radical and concurrently form Mn^3+^ and MnP compound II as an Fe^4+^ oxo-porphyrin complex. The iron bound oxygen and two H^+^ ions react to reduce the MnP compound II. Through this reaction, another Mn^2+^ is oxidized to Mn^3+^, the Fe^3+^ ion is reformed, and one second water is released.

Although Mn^3+^ is unstable in solution, it can be stabilized by forming chelate with carboxylic acids, such as malonate, oxalate, oxaloacetate, malate, and even organic acids derived from glucose or cellobiose oxidation [13]. More importantly, the Mn^3+^-acid chelates have a higher redox potential and can penetrate through the barrier of lignocellulose, thus facilitating degradation of recalcitrant non-phenolic lignin. MnP is inhibited by NaN_3_ and ascorbic acid, but stimulated by GSH (glutathione) and unsaturated fatty acids, such as Tween 80, by forming highly reactive sulphor and peroxyl radicals, respectively [14,15]. Like Mn^3+^-acid chelates, these radicals can also move far away and infiltrate through lignocellulose and attack non-phenolic lignin. For example, GSH stimulates oxidation of the benzyl methyl group in the non-phenolic lignin, veratryl alcohol, and the model dimer compound, which leads to breakage of the ether bond [14]. The wide range of substrate oxidizing capability renders it an interest for biotechnological applications in several industries.

### 2.3. Lignin Peroxidase

Lignin peroxidase is the first lignin-degrading peroxidase ever discovered. Like classical peroxidases, such as the horseradish peroxidase (HRP), LiPs are monomeric hemoproteins, with molecular masses around 40 kDa [16]. In 1983, Tien isolated the first LiP from *P. chrysosporium*, which can degrade model lignin compounds and the natural lignin from spruce and birchwood [17]. LiPs are secreted glycoproteins with heme as the co-factor. Atomic absorption spectroscopy indicates that LiP has ferric, but not copper, zinc, manganese, molybdenum, or cobalt, in the enzyme. The characteristic of atomic absorption spectroscopy of LiP is similar to those of other heme peroxidases, such as horseradish peroxidase and c-type cytochrome, which have one heme constituent in their active center. Although the activity of LiP is dependent on hydrogen peroxide, high concentrations of H_2_O_2_ could be deleterious to an LiP. LiP is most often found in the white-rot fungi, such as *P. chrysosporium*, *Trametes versicolor*, *Bjerkandera adusta*, and *Phlebia radiata*. Note, however, not all white rot fungi have an LiP. Since LiP has a higher redox potential than laccases and MnP [18], it can oxidize both phenolic and non-phenolic substructures of lignin even in the absence of mediators, and therefore appears to be more effective than laccase [19].

Similar to MnP, with two electron oxidation of ferric, LiP produces a compound I intermediate and an oxoferryl iron porphyrin radical cation with the reduction of H_2_O_2_. Then, two consecutive one-electron reductions of compound I take place, firstly yielding compound II and then returning LiP to the ferric oxidation state to complete the catalytic cycle [20].

### 2.4. Versatile Peroxidase

Versatile peroxidase (EC 1.11.1.16) is another member of peroxidase families degrading lignin. The first VP (versatile peroxidase) was discovered in *Pleurotus eryngii* [21], and then expanded in other species, such as *Pleurotus* and in *Bjerkandera*. VP was initially studied as an isozyme of MnP, but researchers quickly realized that VP bears activity that does not depend on manganese. VP can oxidize not only Mn^2+^, but also phenolic lignin and non-phenolic lignin, such as veratryl alcohol [22]. Therefore, VP is regarded as a hybrid of LiP and MnP in terms of biochemical properties. Bearing two active sites, VP can oxidize not only the low-, but also the high-redox-potential lignin directly. The first active site is the exposed heme edge that provides VP the capability to oxidize phenols and dye compounds in the absence of Mn^2+^. The second site is the exposed Trp-164 that is located on the surface of the enzyme [23]. The two sites are reminiscent of those in MnP and LiP, respectively. Interestingly, if Mn^2+^ (the substrate of MnP) and veratryl alcohol (the common used substrate of LiP) are not present in the reaction, VP displays some characters distinct to MnP and LiP, and the degradation is dependent on the concentration of hydrogen peroxide [24]. The catalytic cycle of VP for oxidation of substrates follows a similar mechanism of oxidation as other members of the heme peroxidase family. For Mn^2+^ oxidation, the cation is bound by three carboxylates from glutamate and aspartate residues at a surface close to the heme propionate [25].

### 2.5. Dye Peroxidase

DyPs are a family of heme peroxidases that are unrelated to well-known peroxidases [26]. They have a unique protein structure, as well as a distinct amino acid sequences, divergent from the other class II lignin-degrading peroxidases, with a substrate preference for anthraquinone dyes and high peroxidase activity toward a variety of organic compounds [27]. All DyPs contain an iron protoporphyrin IX (heme cofactor) as a prosthetic group and the NH_2_-GXXDG-COOH motif in their primary sequence as a part of the heme-binding region [26].

The first DyP was discovered in *Geotrichum candidum* Dec 1 with the ability to decolorize 18 synthetic dyes [28]. It was named because of its ability to decolorize various dyes [29]. However, it is obvious that decolorization is not the physiological function of DyPs. Although lignin degradation has been proposed as one of the physiological roles of DyPs, only part of DyPs has or is predicted to have a signal peptide. Therefore, there are still debates about the functions of DyPs since the intracellular DyPs may be involved in metabolism of certain organics other than lignin molecules. DyPs are found in both eukaryotes (fungi) and prokaryotes (bacteria). The bacteria DyPs have been found in species, including *Bacteroides thetaiotaomicron*, *Shewanella oneidensis*, *Anabaena* sp., *Escherichia coli*, *Amycolatopsis* sp., and *Pseudomonas*, and the fungal DyPs have been discovered in *B. adusta*, *Termitomyces albuminosus*, *Irpex lacteus*, *Exidia glandulosa*, and *Mycena epipterygia*. The biochemically analyzed DyPs are characterized by very low optimal pH and preference of anthraquinone substrates.

DyPs are classified into four subfamilies (type A-D). Type A DyPs are extracellular bacterial proteins with a Tat signal peptide. Many bacterial DyPs belong to type B and C, and are predicted to be intracellular. Most fungal DyPs belong to type D. Interestingly, the DyPs from *Rhodococcus josti*, *Amycolatopsis* sp., and *Pseudomonas fluorescens* can oxidize Mn^2+^, which is a characteristic of MnP and VP [30,31,32].

The peroxidative mechanism of DyP was proposed to be essentially the same as other heme-containing lignin-degrading peroxidases [33]. First, H_2_O_2_ enters the heme cavity where it replaces one water occupying the sixth ferric iron coordination site [34]. Then, the heme is oxidized by two electron transfer events to the radical-cationic oxoferryl species, Compound I. DyP extracts two electrons from a substrate and the heme is reduced back to its initial state [34].

## 3. Linking Oxidative Ability of Lignin-Degrading Enzymes to Organics Detoxification

Organics are natural or synthetic chemicals widely used or encountered in the modern world in areas of agriculture, industry, or our daily lives. Indiscriminating and excessive use of artificial organic reagents leads to pollution of the environment [35]. There are lots of such organic chemicals, such as polycyclic aromatic hydrocarbons (PAHs), dyes, and pesticides, in the environment, which have become a major concern for life safety worldwide.

PAHs are toxic pollutants widely distributed on the Earth [36]. They are composed of benzene homologs, which have two or more fusion aromatic rings in a linear, angular, or clustered way, posing serious health risks to animals and humans [37]. Dyes, typically consisted of azo- and anthraquinone-dyes, are a large family of hazardous organic pollutants [38] used in paper production, textile industry, and food technology [39]. Being chemically diverse, most synthetic dyes can lead to many environmental and health problems [40]. In the textile industry, azo-, triarylmethane-, and anthracene-derivatives are commonly used. Pesticides are important chemicals widely used in agriculture [41]. Excessive use of pesticides undoubtedly leads to environmental pollution [35] and, in turn, illness to animals and humans [42]. For example, there has been a growing concern about the production, use, and disposal of substances suspected of interfering with the endocrine system of humans and wildlife. These substances, known as endocrine disrupting compounds (EDCs) include nonylphenol (NP), bisphenol A (BPA), triclosan (TCS), 17a-ethinylestradiol (EE2), and 17b-estradiol (E2), and so on [43]. In many cases, these toxic pollutants are difficult to degrade, and are therefore harmful to the environment and human health. With more than 500 types discovered, mycotoxins, the organic fungal secondary metabolites toxic to human and animals in high concentrations, are a threat to the food and feed industries. They are generated by fungi, e.g., *Aspergillus flavus* and *Aspergillus parasiticus*, and are responsible for a lot of illnesses and disorders in humans and animals [44]. Typically, aflatoxin contamination of feed and food results in significant economic losses [45].

Fortunately, the abilities of various white rot fungi to degrade the organics pollutions have been revealed [46]. It is also frequently noticed that the extracellular ligninolytic enzyme systems of these fungi are directly linked to the degradation of these compounds. For example, the ability of microbes to detoxify organic pollutants is enhanced under culture conditions favorable for mineralization of lignin model compounds, strongly suggesting that the lignin-degrading enzymatic system is involved in transformation of anthropogenic compounds [47].

### 3.1. Laccase

Among the lignin-degrading oxidases and peroxidases, laccase may be the most intensively studied enzyme. With great potentials for biotechnological and environmental applications [48,49], laccase is widely distributed in eukaryotic microbes, particularly in those involved in lignocellulose degradation (http://www.cazy.org/AA1.html). It is also frequently discovered in bacteria, such as *Azospirillum lipoferum* [50], Streptomyces [51], Bacillus [52], and Pseudomonas [53].

Laccase extracts one electron from the hydroxyl and amine groups of the benzene ring and passes it to oxygen to form water. Compared to using lignin-degrading peroxidases (LiP, MnP, VP, and DyP), one advantage of using laccase is no need of hydroxyl peroxide as the oxidant, which is both of environmental concern and can inactivate the enzyme at high concentrations. The use of laccase for detoxification started from phenolics [54] and then rapidly expanded to other complex compounds, including azo dyes [55], benzo, pyrene [56], aflatoxin [57], organophosphorus compounds [58], halogenated pesticides [59], and polycyclic aromatic hydrocarbon [60]. Precipitates form during phenolic compounds’ detoxification. This can be explained by laccase extracting one electron from the hydroxyl group, thereby forming radicals, which subsequently polymerize and precipitate [54,61,62]. When the compound is a phenolic azo dye, two electrons are sequentially taken from the hydroxyl group and then a carbonium ion is formed in the oxidation, in which the charge is localized on the phenolic ring carbon bearing the azo linkage [55]. Nucleophilic attack by water results in breakage of the azo linkage. For halogenated pesticides, a similar mechanism results in dehalogenation [63]. A few examples are as following.

Using laccase to decolorize dyes has a lot of advantages, such as low cost and the ability to react with high concentrations of contaminants [64,65]. When the laccase from *Trametes sanguineus* was overexpressed in the fungus, *Trichoderma atroviride*, it removed the endocrine disruptors, benzo[α]pyrene and phenanthrene, efficiently [66]. E1, E2, and EE2 can be removed by a laccase from *Myceliophthora thermophila* [67]. It was also demonstrated that laccase from the white rot fungus, *Coriolopsis polyzona*, could eliminate EDCs (endocrine-disrupting compounds) efficiently at a contact time of less than 200 min by using 3.75 U of laccase activity for BPA (bisphenol A) and TCS (triclosan) and 1.88 U for NP (nonylphenol) [68]. At the same time, β-estradiol can be oxidized by a laccase from *Polyporus versicolor* [69]. *P. pulmonarius* Lac2 was identified as capable of degrading AFB1 and AFM1, with the help of redox mediators [70]. The *Chaetomium* sp. laccase was able to decolorize the anthraquinonic dye RBBR (Remazol brilliant blue R) [71].

One drawback of laccase in detoxification is its low redox potential (0.5–0.8 V), which limits its oxidative ability only to phenolic compounds, but not more recalcitrant non-phenolic chemicals with a higher redox potential. Nevertheless, certain compounds (either naturally occurring or synthetic) that are oxidized by laccases to form stable radicals can diffuse freely and mediate oxidation of high redox potential and more recalcitrant compounds. The use of such mediators largely broadens the substrate scope of laccase [72] as well as improves the efficiency of this enzyme. For example, TsL laccase manifests effective decolorization activity towards eriochrome black T (EBT) and malachite green (MG). The mediators, Violuric acid (VA) and acetosyringone (AS), can be used as good candidates for LMS (laccase mediator system) of TsL in dye treatment [73]. The capacity of a laccase from *Trametes versicolor* to degrade pesticides has been demonstrated. This enzyme can be used for degrading five pesticides, including chlorpyrifos, pyrimethanil, chlorothalonil, isoproturon, and atrazine [74]. Zao et al. [75] reduced the concentration of dichlorodiphenyltrichloroethane (DDT) in impacted soils using a polypore white rot fungi laccase. In the presence of 0.3 U mL^−1^ laccase and 1 mM of mediator HBT (hydroxybenzotriazole), isoproturon was degraded completely within 24 h [76]. The most studied mediators may be ABTS (2, 2′-Azino-bis(3-ethylbenzothiazoline-6-sulfonic acid)) and 1-HBT [77]. However, many other compounds may also serve as potential mediators and different laccases may have their preferred mediators. In addition, although synthetic mediators (such as ABTS and 1-HBT) are often found to be efficient, their prices limit true application. This underpins the importance of looking for natural mediators that are both efficient and easy to obtain. Note, besides the beneficial boosting effect, mediators at a high concentration, which are often used in detoxification, may have detrimental effects on a laccase [78].

### 3.2. MnP

MnP is another important ligninolytic enzyme secreted by many wood decaying fungi. The MnP system has been observed to oxidize phenolic and nonphenolic lignin model compounds [79]. It has been used to detoxify a range of organics, including synthetic dyes [80,81,82,83], herbicides [84,85], polycyclic aromatic hydrocarbons [81,86,87], mycotoxins [88,89], estrogens [90], explosives [91], and antifouling compound [92]. In these achievements, MnP is used in combination with H_2_O_2_, Mn^2+^, and a certain organic acid chelator (such as malonate, oxalte, or α-hydroxyl carboxylic acids). In the presence of H_2_O_2_, MnP oxidizes Mn^2+^ to Mn^3+^, which forms a stable chelate with the organic acid. This chelate has a high redox potential and can move freely to oxidize the substrate. Like laccase, the oxidative activity of MnP can be largely stimulated by addition of an appropriate mediator. Glutathione (GSH) and unsaturated fatty acids are two widely used mediators that can boost MnP’s activity in either degrading lignin or detoxification [85,87,89]. In some cases, when mediators are involved, H_2_O_2_, but not Mn^2+^, can be omitted from the reaction system. This has been ascribed to either auto-peroxidation of the unsaturated fatty acids or the trace Mn^3+^ present with Mn^2+^, which reacts with malonate to initiate the reaction cascade [93].

MnP could be used to degrade persistent organic pollutants-PAHs [86,94]. In addition, MnP from *P. ostreatus* could detoxify aflatoxin B1, whose efficiency depends on the enzyme concentration and incubation period [88]. MnP is also a potent bio-catalyst for dye decolorization [95], such as Poly R-478 [96]. In one study, the decolorization of three different dyes-Reactive Red 195A, Reactive Blue 21, and Reactive Yellow 145A-by MnP from *Ganoderma lucidum* IBL-05 was monitored. In 12 h, the decolorization efficiencies for all the tested dyes were 78.6–84.7% [97]. In another investigation, the ability of purified MnP from *Trametes* sp. 48,424 to decolorize dyes was studied. The tested dyes, including Indigo Carmine (indigo dye), Remazol Brilliant Blue R (anthraquinone dye), Remazol Brilliant Violet 5R (azo dye), and Methyl Green (triphenylmethane dye), could be efficiently decolorized by MnP from *Trametes* sp. 48,424 [81].

With these achievements, it should be also noted that high concentrations of Mn^2+^ may not be best for MnP’s performance. In decolorization of a highly recalcitrant polymeric dye (Poly R-478), 33 μM of Mn^2+^ is much better than 1 mM. It can be inferred that there is an optimal combination of the concentrations of Mn^2+^ and H_2_O_2_ [98]. This is important since the usage of high concentrations of manganese is also an environmental threat. The importance of the organic acid for MnP’s activity has been elucidated in a paper regarding lignin and dyes’ degradation by two MnPs from *I. lacteus* [99]. In this regard, malonate is the organic acid most widely used. However, in one case, malonate was found to inhibit detoxification of Orange II by the MnP from *Bjerkandera* sp. (BOS2) [100]. Therefore, the acid chelate should also be carefully tested for the organics to detoxify.

The synergy of MnP with laccase or other peroxdases has not been systematically studied. However, it is noticed that under natural conditions, fungi generally secrete more than one oxidase or peroxidase for oxidation of lignin or pollutants. It is also well-known that accessory enzymes, such as glucose oxidase or alcohol oxidase, can provide hydrogen peroxide to MnP by oxidizing its appropriate substrate, such as glucose. It has been presented that the purified laccase and MnP from *T. versicolor* have only additive effects in decolorization of an azo dye amaranth [101]. However, a laccase from *Stropharia rugosoannulata* has the ability of oxidizing Mn^2+^; and in presence of malonate, it can provide hydrogen peroxide for MnP and initiate the peroxidation [102]. In another trial, the MnP from *Dichomitus squalens* also exhibits a synergistic role with the laccase-1-HBT system in decolorization of an azo dye Reactive Orange 16 [103]. These demonstrate that MnP and other lignin-degrading enzymes can cooperate in detoxification.

### 3.3. LiP

The first lignin peroxidase discovered in *P. chrysosporium* can oxidize both phenolic and non-phenolic aromatic compounds due to its high redox potential [17,104]. Thereafter, LiP-expressing basidiomycetes are frequently explored for detoxification, particularly for phenolic compounds-bearing wastes, such as the effluents from paper-pulp mills [105]. LiP by itself catalyzes polymerization of phenolic compounds, regardless of their origin from the constituents of lignin or effluent of olive oil factories [104,106]. Dimers, trimers, and tetramers are found in LiP-treated reactions containing these chemicals [107]. Through polymerization, the phenolic compounds decrease their reactivity and solubility and tend to precipitate, thereby alleviating the toxicity [108]. It is also often noted that phenolic pollutants can be completely mineralized by the LiP-producing microorganisms, suggesting that other enzymes in addition to LiP are involved [109].

LiP has been used in treating paper mill waste and also in detoxifying delignified feedstock for biorefineries [110], halogenated phenol toxics [108], and endocrine-disrupting pollutants (*p*–*t*-octylphenol, bisphenol A, estrone, 17 β-estradiol, and ethinylestradiol, which all have phenolic hydroxyls) [111]. Decolorization of synthetic dyes is also reported. In one case, up to 40% of methylene blue was removed by addition of ~0.5 U/mL of LiP from *P. chrysosporium* [112]. In another case, after 1 h treatment by Lip from *S. griseosporeus* SN9, the color removal rates were 18%, 35%, and 69% for RBBR, AB, and CBB, respectively [113]. When the *G. lucidum* IBL-05 LiP was entrapped in Ca-alginate beads, the decolorization of Sandal reactive dyes were in the range of 80–93% [114]. Ollikka et al. [115] investigated the ability of some lignin peroxidases from *P. chrysosporium* to decolorize azo, triphenyl methane, heterocyclic, and polymeric dyes. The capability of lignin peroxidase to decolorize these dyes in the presence of veratryl alcohol as a mediator was confirmed. LiP has been demonstrated to be capable of directly oxidizing PAHs [116]. The LiP of *P. chrysosporium* can degrade the herbicide isoproturon [117] and BPA (46.1%) [111].

One prominent feature of peroxidases is that their ability to oxidize substrates can be largely affected by chemical compounds supplemented in the reaction system. LiP, as a member of the class II peroxidase, is not an exception. Oxidation of 4-bromophenol by an LiP from *P. chrysosporium* Burds BKM-F-1767 is enhanced by the non-phenolic substrates, veratrole and 3,4-dimethoxycinnamic acid; gelatin; and the co-substrate, syringaldehyde [107]. Repression of H_2_O_2_-dependent inactivation of LiP by gelatin and reverting of LiP to its native state are responsible for improved oxidation. Apparently, this stimulatory effect is highly dependent on enzyme, substrates, and the compounds used. For example, veratryl alcohol does not have any positive effect in decolorization of methylene blue by LiP [112]. Moreover, ferulic acid and caffeic acid even suppress oxidation of 4-bromophenol by the LiP from *P. chrysosporium*. The LiP-mediated degradation of 2,4,6-trinitrotoluene (TNT) is inhibited by the reaction intermediate, hydroxylamino-dinitrotoluene [118].

### 3.4. VP

Direct or indirect involvement of versatile peroxidase in degrading phenolic and non-phenolic pollutants, lignosulfonates, and xenobiotics has been discovered for many lignin-utilizing basidiomycetes [119,120]. As a hybrid of MnP and LiP, VP has been found in *Pleurotus eryngii*, *Bjerkandera* sp., *P. pulmonarius, P. ostreatus*, and *B. adusta*. In ligninolysis, VP uses two strategies in catalysis, one through direct contact of lignin and another through oxidation of Mn^2+^ to Mn^3+^, which diffuses and penetrates the embedded inaccessible lignin component. Both mechanisms can be responsible for organics’ detoxification, depending on the nature of the organics.

The VPBF from *Bjerkandera fumosa* can effectively oxidize polycyclic aromatic hydrocarbons containing three and four aromatic rings in the presence or absence of Mn^2+^ [121]. The six tested PAHs are anthracene, phenanthrene, fluorene, pyrene, chrysene, and fluoranthene, varying in ionization potential, solubility, and number of aromatic rings. A VP from *B. adusta* UAMH 8258 can degrade three halogenated pesticides (bromoxynil, dichlorophen, and pentachlorophenol) among 13 tested. These pesticides have a common feature of bearing phenolic hydroxyl groups. Interestingly, Mn^2+^ inhibits rather than stimulates oxidation of the three pesticides possibly because of binding competition [122]. Unsaturated fatty acid (such as Tween-80) largely stimulates deconstruction of these PAHs due to an interplay of Mn^3+^ with Tween-80 and subsequent generation of free radicals. It was proved that VP4 from *Pleurotus ostreatus* can decolorize the azo dyes, Orange II, Reactive Black 5, and Amaranth [123]. The concentrations of Mn^2+^ and malonate collectively determine the removal rate of these compounds, corroborating the importance of MnP rather than LiP activity [124].

Research has focused on the optimization of detoxification conditions for five endocrine disrupting compounds (bisphenol A, triclosan, estrone, 17 β-estradiol, and 17 α-ethinylestradiol), which all have a phenolic hydroxyl group. Eibes et al. investigated the degradation of estrogen hormones by VP from *Bjerkandera adusta*. The results showed that estrogens were completely degraded within several minutes [125]. In a more recent study, nonylphenol was removed by VP from *Bjerkandera* sp. [126]. In addition, purified VP from *Bjerkandera adusta* was able to decolorize dyes, including azo, phthalocyanines, and anthraquinones [127].

In analyses using isothermal titration calorimetry for humic substrates, VP-BA from *B. adusta* displays synergy with LiP and MnP when both enzymes are active. This is thought to be ascribed to cooperative allosteric conformational change [128]. One prominent phenomenon that may be overlooked is that class II peroxidases, such as a VP, from *B. adusta* can cooperate with laccase to delignify plant biomass as well as detoxify biorefinery wastewater [129].

### 3.5. DyP

The DyP enzymes are a special kind of lignin-modifying enzymes. The majority of DyPs are found in bacteria, among which most are predicted to be intracellular, suggesting that their physiological role is involved in the metabolism of certain compounds. Nevertheless, more and more extracellular DyPs are discovered to be able to oxidatively degrade lignin model compounds and even large polymeric lignin, pinpointing their participation in lignin deconstruction. Generally, DyP has a heme prosthetic group and two catalytic sites, with one located near the heme co-factor and one being the surface protein radical.

DyP is outstanding for its ability to decolorize dyes. Indeed, the first DyP identified can degrade 18 different kinds of synthetic dyes [28]. While the substrate spectrum of DyP is steadily increasing, anthraquinone dyes appear to be the most favoured substrates for biochemically characterized DyPs. Azo, di/tri-arylmethane, xnthene, indigoid, carotenoid, and pathalocyanrine dyes are now discovered to also be substrates of DyPs [130]. Interestingly, some DyPs need assistance from other enzymes to completely bleach dyes while other enzymes do not have this requirement.

DyP can be a useful biocatalyst for environmental applications because of their ability to degrade dyes, indicating that the VP can be used for the bioremediation of contaminated water [26]. Research has shown that the *Irpex lacteus*-DyP4 is able to oxidize most of the anthraquinone dyes, azo dyes, phenazine dyes, triphenylmethane dyes, and aniline dyes. The maximum efficiency of *Il*-DyP4 to decolorize Poly R-478 at an enzyme concentration of 100 nM was 15.76% [131]. DyP from *Vibrio cholerae* can degradate anthraquinone dyes, such as reactive blue 19 [132]. DyP from *Saccharomonospora viridis* DSM 43,017 could decolorize several triarylmethane dyes, anthraquinonic, and azo dyes under neutral to alkaline conditions efficiently [133]. The crude rDyP from *Aspergillus oryzae* has a decolorization ratio of more than 94% [134].

In addition to the promiscuous substrate specificities, some DyPs are impressive for their ability to oxidize Mn^2+^ [32]. This trait can be ascribed to a small Mn^2+^-binding pocket near the edge of heme. Mutation of the distal heme residue, Asn246, in the *R. jostii* RHA1 DypB dramatically improves the Mn^2+^-oxidizing ability by 80- and 15-fold for the apparent *k*_cat_ and *k*_cat_/*K*_m_ values, respectively [30]. It is noteworthy that in the presence of Mn^2+^, the N246A mutant can oxidize and transform hard wood kraft lignin. The Mn^2+^-oxidizing ability could be harnessed in the detoxification of organics with high redox potential. However, this trait has not yet been explored in detoxification.

One advantage of using DyP, but not class II peroxidases, for detoxification may be the comparably easier heterologous expression of DyP. Many bacterial DyPs can be conveniently expressed in *E. coli*. DyP can even be expressed in a soluble apo-form, and subsequent addition of heme restores its activity. An additional feature of DyP is its preference for acidic pH. Many DyPs perform best at ~pH3. Unexpectedly, DyP is actually not so stable at an acidic pH. In contrast, it is much more stable at higher pHs. Besides, substrate-dependent changes of pH profiles have been discovered for a DyP from *Bacillus subtilis* [135].

## 4. Future Perspectives

By degradation and mineralizaion of lignin, ligninolytic enzymes play an important role in the carbon cycle of lginocellulose. The low substrate specificity, which may cast a problem for other highly specialized enzymes, becomes the basis for their utilization as a versatile biocatalyst in organics’ detoxification. Laccase and lignin-degrading peroxidases have been evidently demonstrated as powerful biocatalysts for detoxification of organic pollutants, and, therefore, have wide application potentials in textile, food, feed, and environmental industries. The present review thus provides recent progresses in linking the oxidative activity of these lignin degrading enzymes to organics’ detoxification.

Although there has been numerous research on enzymatic degradation of lignin as well as organics’ detoxification, due to the large variation in the type and structure of lignin and organics, the related mechanisms are not understood entirely [136]. However, we may still find common and different features in these reactions from the perspective of biochemistry. On the one hand, laccase can directly oxidize phenolic lignin model compounds without assistance of small molecule mediators. Oxidized lignin tends to polymerize, underlying its function in directing lignin polymer biosynthesis. MnP and some DyP are also able to directly oxidize phenolic compounds. Similarly, the laccase-treated phenolic organic pollutants tend to form large polymers, which will precipitate, thus alleviating the toxicity of these organics. For non-phenolic lignin compounds with high redox potential, laccase and MnP react with mediators to form free radicals, which have high redox potential and will in turn attack lignin. This characteristic is widely employed for organic detoxification, with the generated free radicals aiding in modifying or breaking the recalcitrant covalent bonds in the organic pollutants. On the other hand, there is obvious differences in the reactions of ligninolytic enzymes with lignin and organics. The mediators used by laccase and MnP are normally small metabolites of the microbes and the living organisms in their natural habitats. For example, in *Pycnoporus cinnabarinus*, 3-hydroxyanthranilate, a natural pigment precursor, acts as a mediator in the degradation of the lignin model compound [137]. Oxalate, the excreted metabolites of *Irpex lacteus,* which is synthesized from glyoxylate dehydrogenases and oxaloacetases, can chelate unstable Mn^3+^ ion for higher performance of the ligninolytic enzymes [138]. In contrast, in the detoxification of organics using ligninolytic enzymes, it is not necessary to be limited to using natural mediators, but one can screen a range of artificial or natural mediators for the highest performance of the enzymes. However, in certain specific cases, such as mycotoxin detoxification, natural mediators may still be favored. We also notice that, in nature, ligninolytic enzymes are usually produced by microbes as an enzyme cocktail containing two or more kinds of the laccase and class II peroxidases, which may or may not have a synergistic role in the degradation of lignin. However, most current research focuses on using only one kind of the ligninolytic enzymes to detoxify organics.

With these pieces of knowledge in mind, we realize that there is still a long way to go to make full utilization of these enzymes.

Firstly, among the discussed enzymes, laccase is the one that can be most easily produced at a large scale. Nevertheless, normally, laccase cannot degrade compounds with high ionization potential. The lignin-degrading peroxidases are better at destructing high redox potential compounds, but they are notoriously difficult to produce heterologously, possibly due to the existence of a heme group as the co-factor. Methods need to be developed, either engineering higher catalytic ability into laccase or generating host strains that can be used to express these peroxidases in large quantity.

Secondly, fragmental pieces of evidence show that laccase and peroxidases can act synergistically in detoxification. Are there more cases of synergy and what are the underlying molecular mechanisms? The mechanistic knowledge can be harnessed to design more powerful detoxifying enzyme cocktails. In another aspect, the components in the reaction system, particularly the mediators, should be systematically investigated for detoxification of a specific compound. Although a lot of chemicals, either artificially produced or naturally occurring, can serve as a mediator, the best mediator should be the one that can largely speed up the reaction while not bringing any new pollution into the environment.

Thirdly, the use of lignin-degrading enzymes for detoxification has rapidly expanded from dyes to other toxic compounds. While dye decolorization is easy to monitor, detecting the degradation of other chemicals is more difficult, time-consuming, and equipment-dependent. Currently, the ability of an enzyme to detoxify is often explored with trial-and-error, which is unpredictable with a large uncertainty. Moreover, detoxification is normally in harsh conditions, requiring long durability of enzymes, which necessitates engineering of enzymes for higher stability. Therefore, if a chemical could be identified with a change of ease-of-monitoring (such as color) proportional to the ability of enzymatic detoxification, then this compound can be used as an indicator, which will undoubtedly speed up the discovery and engineering of novel, powerful detoxification enzymes.

## Abbreviations

CAZy	Carbohydrate-Active enzyme
AA	Auxiliary Activity
LiP	Lignin Peroxidase
MnP	Manganese Peroxidase
VP	Versatile Peroxidase
DyP	Dye-decolorization Peroxidase
GSH	Glutathione
ABTS	2, 2′-Azino-bis(3-ethylbenzothiazoline-6-sulfonic acid)
1-HBT	1-Hydroxybenzotriazole
H_2_O_2_	Hydrogen Peroxide
